# Optical Methods of Methane Detection

**DOI:** 10.3390/s23052834

**Published:** 2023-03-05

**Authors:** Mirosław Kwaśny, Aneta Bombalska

**Affiliations:** Institute of Optoelectronics, Military University of Technology, gen. Sylwestra Kaliskiego 2, 00-908 Warsaw, Poland

**Keywords:** methane, detection, gas sensor, optical methods, concentration

## Abstract

Methane is the most frequently analyzed gas with different concentrations ranging from single ppm or ppb to 100%. There are a wide range of applications for gas sensors including urban uses, industrial uses, rural measurements, and environment monitoring. The most important applications include the measurement of anthropogenic greenhouse gases in the atmosphere and methane leak detection. In this review, we discuss common optical methods used for detecting methane such as non-dispersive infrared (NIR) technology, direct tunable diode spectroscopy (TDLS), cavity ring-down spectroscopy (CRDS), cavity-enhanced absorption spectroscopy (CEAS), lidar techniques, and laser photoacoustic spectroscopy. We also present our own designs of laser methane analyzers for various applications (DIAL, TDLS, NIR).

## 1. Introduction

Methane is one of the gases that is most frequently analyzed, and its detection has many issues. The first is safety in the mining area, where methane accompanies coal deposits. About 4.5–15% of the concentration of methane in the air forms an explosive mixture and can cause explosions in hard coal mines. It is therefore very important to detect concentrations that are much lower than those that can cause explosions due to the time needed to evacuate the affected area. The problem with methane sensors currently used in mines is the phenomenon of saturation and aging of pellistor sensors. These result in a limited time to operate modern methanometers and the need to frequently calibrate them.

Another issue with the presence of methane in the environment is the monitoring of landfills, where methane appears as a result of anaerobic digestion processes. Methane is also obtained via fermentation in biogas plants, where the gas obtained in this process is used directly or further processed into methanol and used as fuel.

In the area of fuel applications, methane is the main component of compressed natural gas (LNG), which is increasingly being used as a green automotive fuel. The concentration of methane in LGN ranges from 70 to 95%. Natural gas is widely used in households and industries.

The development of methane gas leak detectors is then crucial to provide a useful tool to detect faulty natural gas pipes. Methane is also a powerful greenhouse gas (GHG) with a global warming potential; it was 28 times greater than carbon dioxide (CO_2_) over a 100-year period [1] and it significantly contributes to climate change [2]. The atmospheric methane concentration has increased dramatically from approximately 800 parts per billion (ppb) in the early 1900s to 1800 ppb in 2016 [3].

In 1988, methane clathrates (methane hydrate, methane ice, methane hydrate), crystalline substances composed of water molecules (85%) and methane (15%), were discovered. In the 1960s, hydrate deposits were found in Siberia and in the next decade, significant amounts of them were discovered on the shelves of the oceans. Approximately 170 L of methane gas can be extracted from one liter of methane hydrates (in terms of normal conditions).

Formisano et al. [4] reported that methane has been detected in the atmosphere of Mars, with an average concentration of 10 ± 5 parts per billion by volume (ppbv) and varying from 0 to 30 ppbv across the planet. The measurements were made using a planetary FTIR spectrometer onboard the Mars Express spacecraft. The presence of this gas theoretically increases the chances of finding traces of life on the Red Planet. Methane is dying very quickly in various ways in the Martian atmosphere. This is why significant clouds of this gas in the northern hemisphere of Mars indicate there is a continuous release of methane.

Trace amounts of methane detected are also used in medical diagnostics. When there is an imbalance in the microscopic gut flora, irritable bowel syndrome is formed and methane appears in the breathed air. Sugars that are not digested before reaching the large intestines are processed by microbes. This leads to the formation of methane which is absorbed into the bloodstream and then transported to the lungs [5].

## 2. Methane Detection Methods

In the eighteenth century, the causes of explosions in mines and how to counteract them were not known, even though mining has existed since ancient times. The first indicator that informed miners about the threat of explosions was the so-called Davy lamp. In every mine, fresh air with adequate oxygen content must flow in for people to breathe freely. At the same time, carbon dioxide and methane are produced. Each ton of coal can contain up to 30 m^3^ of methane. At the right concentration, as methane comes into contact with a flame, an explosion might occur. Both gases are odorless, and the danger cannot be smelled or seen. It is impossible to work without lighting in mines, which are electric today, but were once flame lamps. The famous English scientist, Sir Humphry Davy, constructed a mining indicator gasoline lamp to detect methane. If there was methane in the surrounding air, its flame turned more and more blue. The invention entailed using the good thermal conductivity of metals. The flame of the lamp and the gases escaping from it had a high temperature, which could cause an explosion when in contact with mine gases. The flame of the lamp was surrounded by a double metal mesh with small holes, preventing it from getting outside. Thanks to its good thermal conductivity, the metal cooled the flame gases, lowering their temperature below the ignition point of the mine gas. The lamp went out when the carbon dioxide content increased to 5–6% and the oxygen content decreased to 17%.

Various types of sensors are used to detect methane, such as: optical sensors, calorimetric sensors, pyroelectric sensors, semiconducting metal oxide sensors, and electrochemical sensors [6]. Catalytic (thermometric) and thermal conductivity sensors are the most commonly used for detecting methane, which are increasingly being replaced by optical ones. The Thermal Conductivity Detector (TCD), also known as katharometer, is the oldest type of sensor used to study the composition of gas mixtures. It works by measuring the difference in the thermal conductivity of a carrier gas, caused by a change in its chemical composition. The sensor consists of two or four fibers, or thermistors, connected to form an unbalanced Wheatstone measuring bridge and it is heated by a current flowing through them. The sensor is characterized by high sensitivity, linearity of response over a wide measuring range, and simple design. Its main disadvantages are the lack of selectivity and the need to ensure constant reference conditions, a constant sensor temperature, and constant gas flow rate.

Catalytic sensors have been used since the 1920s to detect methane in mines and have remained the most popular type of sensor used in mining. The sensors consist of two elements: active and passive elements. On the active element, a catalytic combustion reaction takes place, while the passive element serves as a reference to compensate for external changes in temperature, pressure, and humidity. The sensor is incorporated into the Wheatstone measuring bridge. Currently, two types of catalytic combustion sensors are used: hot fiber (hot wire) and pellistors. The first is made of hot platinum fiber placed directly in a stream of a mixture of combustible gas and air. The platinum wire plays a triple role: it acts as the catalyst for the combustion process, heating element, and resistance thermometer. The electric current flowing in the circuit heats both pellistors to a temperature of 400 °C to 500 °C, at which time catalytic oxidation of the gas mixture can occur.

Catalytic (thermometric) thermal conductivity sensors are increasingly being replaced by optical ones. Modern methods for detecting methane in the air can be divided in many ways, due to the place of measurement—the analysis of samples in stationary conditions in laboratory rooms, or continuous monitoring in the open area. To analyze the samples collected “in situ”, analytical techniques with very high sensitivity are used, such as mass spectrometry, ion mobility spectrometry, or gas chromatography. For continuous measurements in the field, optical methods are basic. Optical methods have many advantages: they allow for the remote measurement of methane and measurements of extremely low concentrations at the level of ppm, ppb, and ppt. Their main limitation is the overlapping absorption bands of other hydrocarbons and water. In this review, we present various optical techniques for detecting methane: non-dispersive infrared (NIR) technology, direct tunable diode spectroscopy (TDLS), cavity ring-down spectroscopy (CRDS), cavity-enhanced absorption spectroscopy (CEAS), lidar techniques, and laser photoacoustic spectroscopy. There are numerous laboratory studies on the methods for detecting methane. Our goal was to analyze the instruments that have found practical applications.

## 3. Methane Absorption Spectra

Optical gas detection using absorption spectroscopy is based on the Lambert–Beer law (1,2):

(1) $$I\left(\lambda \right)=I_{0}\left(\lambda \right)\exp\left[-a\left(\lambda ,C\right)\cdot L\right]\;\;\;\;a\left[cm^{-1}\right]$$

(2) $$I\left(\lambda \right)=I_{0}\left(\lambda \right)\exp\left[-\alpha \left(\lambda \right)\cdot C\cdot L\right]\;\;\;\;\alpha \left[ppm\cdot cm^{-1}\right]$$

where: *I*—light intensity transmitted by the medium with the gas, *I*_0_—intensity of light incident on the medium, *C*—concentration, a, *α*—absorption coefficients, and *L*—optical path length, gas concentration.

**Figure 1:**
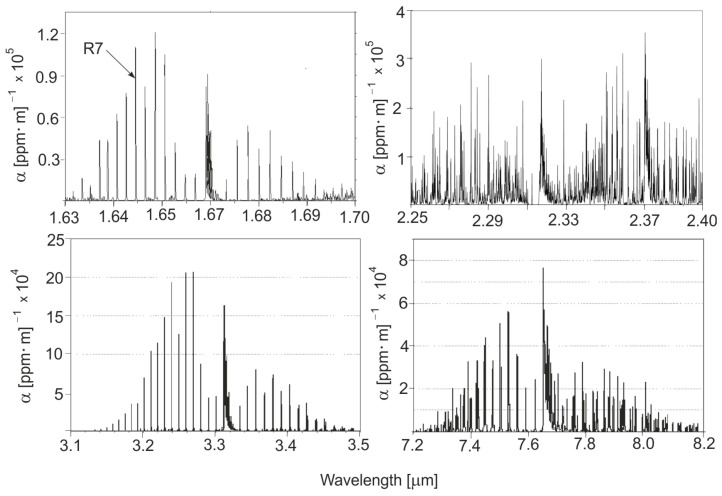
Methane absorption spectra. The data were compiled from the HIRTRAN database.

Quantitative gas spectra are available at the US National Institute of Standards and Technology, Pacific Northwest National Laboratory (PNNL) [7] and can be calculated from the HITRAN database [8]. The optical methods for methane detection use its absorption characteristics in the infrared range. Figure 1 shows the methane absorption spectra. The strongest bands occur in the area of deformation vibrations and then valence; they are weaker in the range of overtones.

Methane has many absorption lines in the range of 1.63–1.69 µm. These lines are sufficiently strong with a total absorption (cross-section) of 5.13 × 10^−20^ cm^2^. However, in this band, there are bands of water with a total absorption of 4.34 × 10^−23^ cm^2^ and carbon dioxide with a total absorption of 4.95 × 10^−23^ cm^2^. Assuming that the water vapor concentration is at the level of 1% (10,000 ppm) and CO_2_ is at the level of 0.03% (300 ppm), the analyzed methane absorption lines should be carefully selected so that they are not disturbed by these absorbers. Examples of such lines are R3 (λ = 1.654 µm) and R7 (λ = 1.654 µm). An example of the presence of CH_4_ and H_2_O absorption lines next to each other and the selection of the appropriate bands for analysis are shown in Figure 9. An effective way to improve the spectral resolution of the lines, their separation, and reduction in the half-width is the operation of absorption cells under a reduced pressure of 50–100 hPa.

Instruments that use the NIR range (0.8−2.5 μm) do not require expensive, cooled detectors.

The main problem, however, lies in the low gas absorption coefficients in this range because most gases have only overtone and combination bands, which are much weaker than the absorption basebands in the average IR range. However, the sensitivity achieved by the TDLS technique allows for the detection of trace amounts of various atmospheric pollutants.

## 4. Non-Dispersive Sensors

**Figure 2:**
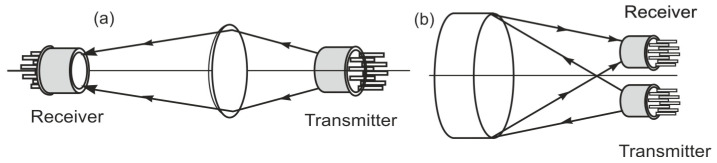
The idea of opto-pair (**a**) with a single pass, and (**b**) a double passage of radiation.

Broadband non-dispersive sensing is one of the simplest techniques used for construction and has the greatest commercial importance. This sensor consists of a combined detector and a light source in a durable housing, which is a flow-through measuring chamber for the gas. Depending on the size of the concentrations and absorption coefficients of the analyzed impurities, cuvettes with different optical path lengths are built. An optical sensor called opto-pair (optoelectronic pair) is shown in Figure 2.

**Figure 3:**
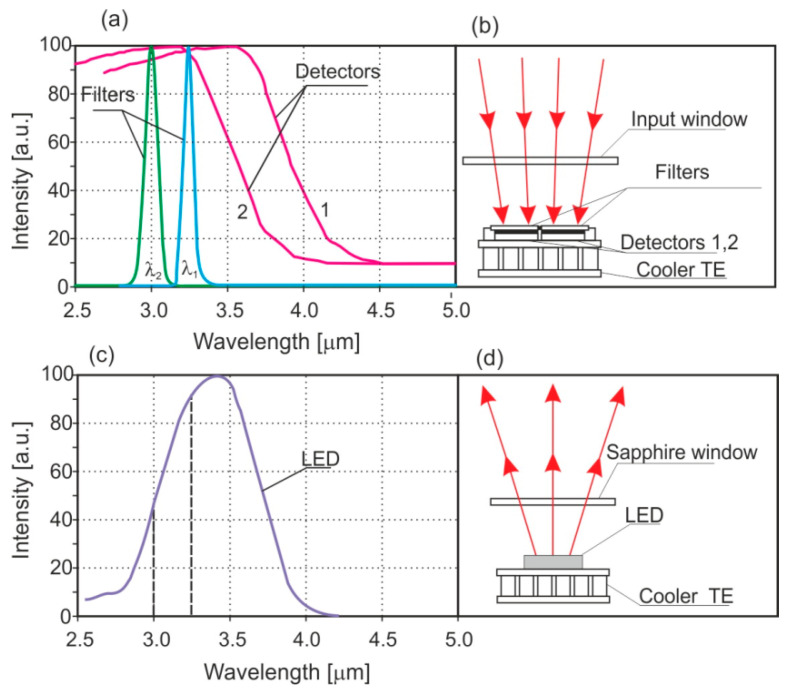
The principle of methane measurement with opto-pair. (**a**) transmittance of filters and the sensitivity of detectors, (**b**) block of detectors, (**c**) source emission spectrum, (**d**) light source block.

Figure 3 shows the differential principle for measuring the methane absorption in an opto-pair sensor. The optical filter of the working channel transmits radiation in the range of 3.23 μm while that of the reference channel transmits in the range of 3.0–2.9 μm. In the 3.0–3.5 µm range, there is no absorption of CO_2_ and H_2_O. This part of the methane spectrum is shown in Figure 1.

The emitted radiation light source passes through a measuring cuvette of the diffusion type. It is divided into two beams and analyzed by detectors in the range of absorption and reference bands. A differential, two-channel measurement eliminates the influence of water and other factors, and the miniature TE cooler provides cooling and stabilizes the temperature up to 0.1 °C. The RS-485 digital output allows for the construction of a spatial control network containing up to 128 m. Compared to similar devices, it is characterized by high measurement stability and low drift. The operation of the device is guided by a microprocessor, which forms a sequence of subsequent pulses for the photodiode controller, and digitally analyzes the amplitudes of signal pulses coming out of amplifiers of 4 and 5 for the working and reference channels. The microprocessor sets the amplitude of the current, and the photodiode driver protects the amplitude stabilized at a certain level. The infrared photodiode emits radiation covering the absorption bands of the analyzed gases, and the detectors register the radiation in the absorption band (detector 1) and out of the band (detector 2). The microprocessor calculates the amplitudes of the working and reference pulses. Their mathematical processing and the gas concentration (C) are calculated using Equation (3):

(3) $$C=-\ln\left(I_{p}-I_{0}\right)/L\left[\alpha \left(\lambda_{p}\right)-\alpha (\lambda_{0})\right]$$

where: *L*—optical path length, *I_p_*, *I*_0_—amplitude of the working channel and reference, and α—absorption coefficient for a given wavelength.

Opto-pairs used by RMt Ltd. (Moscow, Russia) for the 3–4 μm range are made of PbSe LED and PbSe detectors. The instrument is characterized by the absence of moving parts, high mechanical stability, shock resistance, high selectivity, low power consumption, long operating time, reliability, and measurement resolution at the level of 10 ppm (Table 1).

A design of a portable optical sensor for methane detection with a sensitivity of 100 ppm was demonstrated by Aleksandrov et al. [9]. The sensor is based on the use of LEDs operating around 1660 nm and it can operate in environments with a temperature between −20 and 50 °C. The systems use two detectors with optical filtering and the temperatures of the source and detectors are stabilized.

Another example of sensors made of opto-pairs in the 3.5 mm wavelength range is shown in the article of Vargas-Rodrıguez and Rutt [10]. InAsSb(P)- and InGaAs(Sb)-based LEDs were used as radiation sources. The advantages of novel analyzers are their compact size, fast response, and low energy consumption.

Previous non-dispersive optical methods for detecting methane were based on the absorption of infrared radiation in the 3.2–3.4 or 1.65–1.67 μm bands using narrowband interference filters. Instruments based on opto-pairs or photoacoustic phenomena are sensitive to the presence of other hydrocarbons.

**Figure 4:**
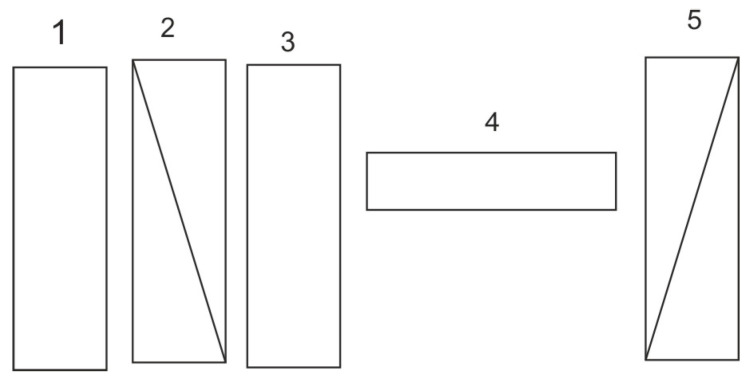
Interference and polarizing filter as an optical correlator: 1—interference filter; 2, 5—crossed polarizers; 3—quartz plates; 4—polarizing modulator.

A much greater selectivity of methane determination can be obtained by means of an optical correlator that reproduces the structure of gas absorption lines [11]. This technique is described in detail by Wojtas et al. [12]. If it is possible to obtain a change in the frequency shift between the filter and the gas absorption line at the detector output, the so-called autocorrelation function can be obtained. Such filters include oscillating mechanical masks, Fabry–Perot interferometers, and interference-polarizing filters (CIPS). The optical scheme is shown in Figure 4.

Using this technology, CIPS analyzers were built at IOE WAT to monitor potential methane leaks near transmission lines and urban areas. The heart of the device is an interference-polarizing filter that consists of an interference filter (1), a pair of crossed polarizers (2,5), phase quartz plates (3), and a polarizing modulator placed between them (4). By selecting the phase thickness of the plate, it is possible to obtain a structure equal in distance to the absorbing filter, which is equal to the distance between the absorption lines.

The radiation of the source passes through the measuring cuvette where the measured gas is pumped, and then through an electronically controlled spectral filter. The transmission spectrum of the filter is matched to the absorption spectrum of the gas being measured. The radiation falls on a photodetector with thermoelectric cooling. The transmittance of the spectral filter and the overlap with the absorption spectrum depend on the applied electrical signal. A signal with different modulation frequencies is created on the receiver. The modulation amplitude and the gas concentration are described by the following Equation (4):

(4) $$\frac{I_{p}-I_{0}}{0.5\left(I_{p}+I_{0}\right)}=1-exp-\left[K\left(\lambda_{p}\right)-K\left(\lambda_{0}\right)\cdot C\cdot L\right]$$

where: *K*—gas absorption coefficient for a specific wavelength, *L*—optical length of the cuvette, *C*—the measured gas concentration, *Ip*—amplitude of the signal on the detector when the position of the electrically controlled spectral filter corresponds to the spectral region of gas transmission, and *I*_0_—the signal on the detector at the moment of position mismatch.

**Figure 5:**
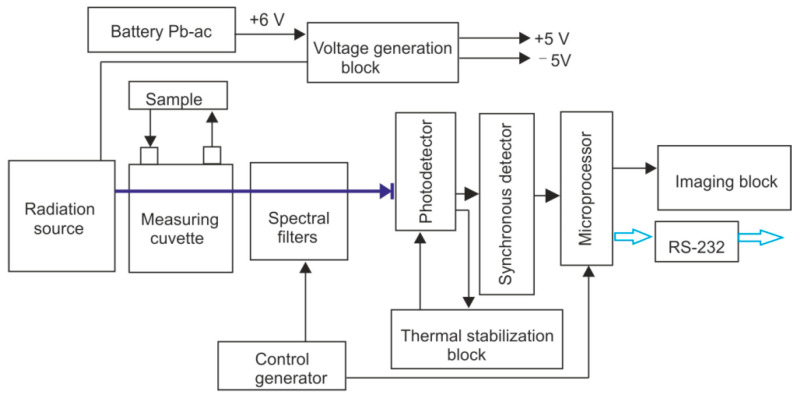
A functional diagram of CH_4_ analyzer.

A functional diagram of the CH_4_ analyzer is shown in Figure 5.

The radiation source is powered from a stabilized source that produces voltage. The operation of the device is guided by a microprocessor, which generates pulses that determine the rhythm of the system for controlling the control generator. The microprocessor produces a digital record of the amplitude of signals coming out of the synchronous detector block. The control generator ensures the generation of voltage necessary for electronic control of the spectral filter and ensures the rhythmicity of the synchronous detector block. An interference filter is mounted directly into the body of the photodetector in the range of 3.4 μm. The temperature of the detector is stabilized by a Peltier plate. The control signal of the thermal stabilization block comes from a thermoresistor mounted directly into the detector body.

The microprocessor determines the modulation amplitude of the measured signal, performs mathematical data processing, and calculates the result—the concentration of the measured gas. The parameters of the instrument are shown in Table 2.

## 5. Tunable Diode Laser Spectroscopy

Optical gas sensors using laser absorption spectroscopy (LAS) rely mainly on multipass gas cells, optical resonators (cavities), and photoacoustic spectroscopy [13,14,15]. The most developed, universal, and advanced optical method used for gas detection is tunable diode laser spectroscopy (TDLS) [16,17,18]. This method is used for both in situ and remote measurements. To date, trace gas grades can be detected with sensitivity at ppt concentration levels using TDLAS [19,20]. In the TDLA method, the emission wavelength of a narrow line-width laser diode is scanned on a single gas absorption line with very high resolution [21], and the peak absorption of the central line is compared with the zero level on both sides of the line. The TDLS method has the following advantages:

- low minimum detection limits, high signal-to-noise ratio;
- high degree of specificity for the test gas. An example is the detection of methane in the presence of other hydrocarbons [22];
- speed of operation; the wavelength of diode lasers can be modulated at a frequency of 100 kHz and even MHz.

Two different measurement techniques are commonly used in TDL—line scanning and wavelength modulation spectroscopy. A wavelength modulation (or frequency modulation−FM) is used to increase the signal-to-noise ratio and improve the MDL. Distributed feedback (DFB) diode lasers are particularly useful in this regard because they emit a single axial mode output. These techniques use single paths or folded gas cells, such as White or Herriot.

The wavelength of distributed feedback lasers (DFB or vertical cavity surface emitting lasers—VCSELs) is tuned by changing the temperature of the laser cavity with a Peltier element in a few seconds, or more rapidly via the injection current at modulation rates (MHz regime) [23]. Instruments based on both these techniques have been commercialized.

The laser wavelength is frequency modulated on the methane absorption line. The change in frequency makes it possible to measure the first or second derivative of the gas absorption spectrum. For the maximum absorption value, the signals of the first harmonic pass through zero, and for the second harmonic they reach a maximum [24,25].

The WM/FM modulations can be combined with other laser absorption spectroscopy techniques such as CRDS and PAS. Sensors based on TDLAS technology are dependent on the availability of suitable laser sources, such as single diffuse feedback (DFB) laser diodes in the near-infrared range for ~3 μm [26], quantum cascade lasers (QCL) in the mid-infrared range (3.5–24 µm) [27], and interband cascade lasers (ICL) with a wavelength of 3–4 μm [28].

Laser diodes made of lead compounds are in practice large-sized systems and require cooling. Coherent lasers including DFG are systems with an integrated structure, and they cannot generate radiation with a wavelength above 5 µm. At the same time, they have low energy efficiency. OPO lasers, on the other hand, provide maximum power. However, these elements are very complex and expensive. An important stage in the development of spectroscopy was the discovery of cascade lasers, which are characterized by an integrated design, narrow emission lines, and high radiation power. QCL and ICL lasers have revolutionized IR absorption spectroscopy [29].

The first QCL laser was developed in Bell’s laboratory. The emission of photons in this laser is the result of a change in the state of the carrier of one type of electron. The length of the produced wave can be selected by making the appropriate thickness of the layers forming quantum wells and separating their barriers. Currently, commercial cascade lasers are available that emit radiation with wavelengths ranging from 3.5 μm to 24 μm. The most important companies where they are manufactured include Alpes Lasers (St-Blaise, Switzerland), Daylight Solutions (San Diego, CA, USA), Cascade Technologies (Cumbernauld, Great Britain), Nanoplus (Meiningen, Germany), Thales (Paris, France), and Hamamatsu (Hamamatsu City, Japan). The most-developed is the cascade laser technology based on the type-I interband transition in InGaAs/InAlAs heterostructures [30]. 

**Figure 6:**
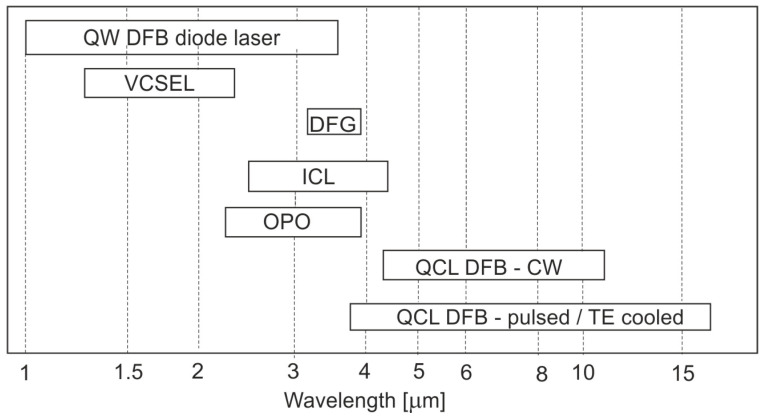
Spectral ranges of radiation sources are used in IR absorption spectroscopy. CW—continuous wave, QW DFB—quantum well distributed feedback, DFG—difference frequency generation, ICL—interband cascade laser, OPO—optical parametric oscillator, and QCL—quantum cascade laser.

The ICL lasers, commercially available since 2009, have revolutionized the detection capabilities of hydrocarbons at the ppb and ppt levels. The range of emission lines for these lasers coincides with the absorption bands in the area of the valence vibrations (3–4 μm) of methane, ethane, ethylene, or acetylene. These sources are characterized by many advantages. They have a small size, can work in either pulse mode or continuous wave (CW) at room temperature and at a low power consumption [31,32,33,34,35]. The spectral ranges of radiation sources in the IR range are shown in Figure 6.

The TDLS method for detecting CH_4_ using NIR LEDs has been used since the 1980s [36,37,38,39,40,41,42,43,44,45,46,47,48,49,50,51,52,53,54]. The introduction of cascade lasers to the market allowed for the construction of CH_4_ sensors with detection sensitivities of ppb and ppt in the ranges of 3.3 µm [55], 7.3 µm [56], and 8.3 µm [57].

One of the leaders in the construction of CH_4_ laser analyzers is Aerodyne Research Inc., USA. This firm has been providing high-precision methane and nitrous oxide measurement instruments to researchers in ecology and agriculture studies of greenhouse gas emissions since 1995. An example is a quantum laser absorption spectrometer (QCLAS) [58]. It was placed onboard the FAAM (Facility for Airborne Atmospheric Measurements), a large atmospheric research aircraft. The source of radiation is a continuous wave, distributed feedback thermoelectrically cooled QCL (Alpes Laser, Switzerland). The laser beam passes through an astigmatic Herritt multipass absorption target (76 m), and falls on a thermoelectrically cooled photovoltaic detector (Vigo Systems, Warsaw, Poland). A QCL laser is scanned over the region of 1275.3–1275.8 cm^−1^ with a frequency of 5 kHz. The laser temperature is maintained at −23 °C using a Peltier cooler. The TDL Wintel software package is used to control the laser operating parameters and calculate the content of methane. The total uncertainty for the methane measurement was ±2.47 ppb. 

**Figure 7:**
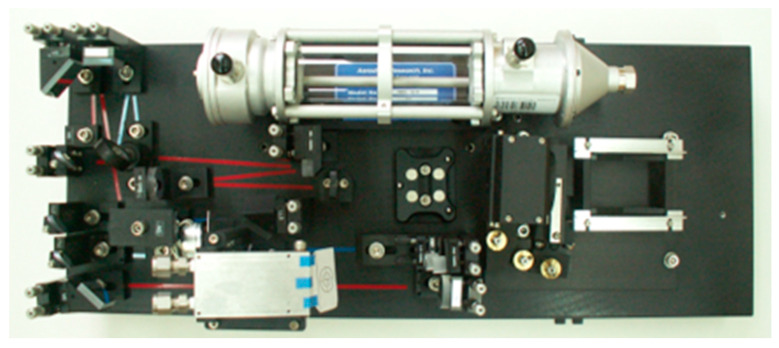
Analyzer view.

At the IOE institute, using components from the company (laser, Wintel software, optics), a similar system was built for measuring the concentration of CH_4_ in various places (around landfills, mines, and pipelines). The system has been efficient and reliable in operation for many years. The basic instrument MODEL QCL-56 (Aerodyne Research. Inc., Billerica, MA, USA) [56] includes a thermoelectrically cooled pulsed laser. The instrument uses a 56-m path, a 0.5-L volume, and a multipass absorption cell in which the gas is kept at a pressure of 30–60 Torr. Infrared detectors are thermoelectrically cooled. The optical components are mounted on an optical contact plate measuring 25 cm × 60 cm (Figure 7). The electronics consist of laser temperature controllers, and current and computerized data acquisition with a speed range from 1 to 20 Hz.

Another example of an analyzer for CH_4_ is based on an interband cascade lasers Compact TDLAS [59]. The instrument consists of two optical cores containing two gallium antimonide-based ICL lasers (GaSb) and a compact multipass gas cell (MPGC). The minimum detection limits for methane and ethane are approximately 5 and 8 ppbv, respectively. Methane concentrations as high as ~5 ppm were detected in the vicinity of the CNG station. The TDLS method was used to build methane analyzers in human breath, using elevated CH_4_ levels as a biomarker of colon fermentation and intestinal problems [60].

The TDLS can be used for breath analysis in clinical diagnosis, therapy monitoring, and for the control of metabolic disorders. 

An example of such an instrument is the integrated laser absorption system (Sensormed) [13]. The analyzer consists of breath collection units, a sample preparation of three biomarker sensors (CH_4_, NO, CO), and a data analysis unit. Single-mode DFB diode lasers with CW tuned to the respective absorption lines of 2.253 mm for CH_4_ and 2.334 for CO were used to simultaneously measure CH_4_ and CO concentrations.

The modulation is carried out using stabilized quartz synthesizers with frequencies f1 = 1 kHz and f_2_ = 1.5 kHz for CH_4_ and CO, respectively. The MUPASS cell consists of two metal mirrors spaced apart with a diameter of 2 inches and a radius of curvature of 2 m each. The measurement accuracy is below 0.1 ppm. The concentration of methane in the breath of a healthy person should not exceed 10 ppm, which means that the accuracy of CH_4_ measurements in the breath is sufficient.

Another system used for the detection of methane, ethane, and formaldehyde in human breath has recently been reported by Winkowski and Stacewicz [61]. In this measurement system, the light source is a tunable ICL CW laser with a power of 30 mW. Changing the wavelength in the range of 3.337–3.334 µm and fine-tuning the laser current in the range of 30–70 mA is achieved using the Thorlabs TLD001 controller (Thorlabs, Newton, NJ, United States). A custom Herriot multipass cell with a 17.5 m path length is used. The change in the laser wavelength is caused by a change in the current, which is modulated with a sinusoidal signal with a frequency of 91 Hz. The laser current is modulated with a sinusoidal signal (91 Hz), which results in the modulation of the laser wavelength. This wavelength modulation is transferred to an intensity modulation which is detected with a PbSe (Thorlabs, PDA20H) (Thorlabs, Newton, NJ, United States) and a lock-in amplifier (Stanford SR830) (Stanford Research Systems, Sunnyvale, CA, United States), which helps to extract the signals at specific harmonics of the modulation frequency. The usable detection level of methane in the system is about 30 ppb [61].

Multiple transition cells are also used in another type of TDLS called intra-pulse spectroscopy using pulsed QCL lasers operating at room temperature [62,63]. The duration of the laser pulse is usually a few microseconds, while the intensity of the laser supply current is several amperes greater than the threshold value. In this way, flat current pulses are obtained. This makes the laser heated locally, and, consequently, the laser frequency moves towards shorter wavelengths. This allows for the laser emission line to be swept around the selected gas absorption line.

**Figure 8:**
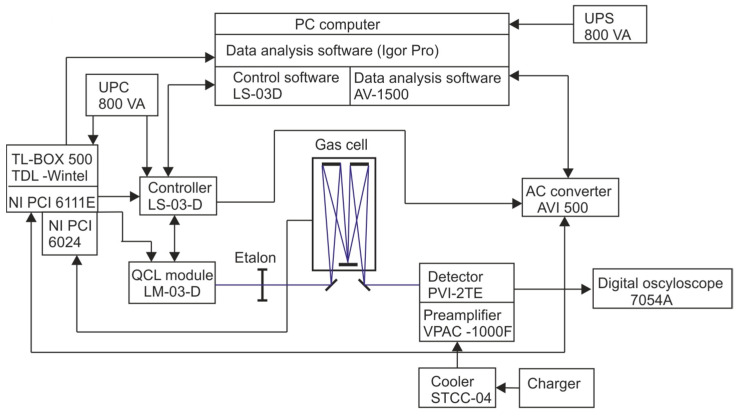
Diagram of the analyzer, view of the LS-03-D controller and the LM-03-D laser module.

This method makes it possible to obtain tuning in the range of up to 6 cm^−1^. The spectral resolution in this case is defined by the instantaneous width of the laser spectrum during frequency shifts. The typical resolution of this method is usually less than 0.01 cm^−1^. The kHz repetition frequencies allow for high fill factors and the use of signal averaging techniques, thus significantly increasing the ratio of the signal power to noise power. During the duration of a long pulse of radiation of the order of 1.5 μs, the temperature of the semiconductor junction increases, which causes the emission to shift towards shorter wavelengths. This allows the laser emission line to be swept around the selected gas absorption line. This technique was commercially introduced by Cascade technologies. IOE built methane analyzers with a QCL laser and a wavelength of 7.85 µm from elements purchased from this company. The diagram of the apparatus is shown in Figure 8.

**Figure 9:**
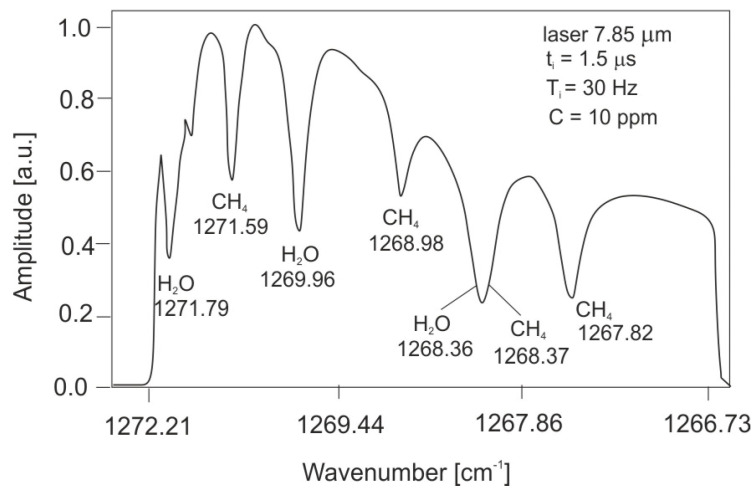
Atmospheric transmission spectrum with methane.

An example of the atmospheric spectrum measured with a cascade laser is shown in Figure 9.

The analyzer uses a professional Herriott-type multiple transition cell with an optical path of 36 m and a volume of 0.5 dm^3^. The laser energy in the pulse is about 150 mW and the repetition frequency is 20 kHz. It enables measurements of methane at the sub-ppm level with a measurement error of about 5% to be obtained.

## 6. Cavity Ring-Down Spectroscopy

The technique of pulsed ring-down absorption spectroscopy (CRDS) was developed by Keefe and Deacon in 1988 [64]. Since the last decade, many different variations of experimental CRDS spectroscopy have been developed [65]. Foltynowicz et al. [66], Berden et al. [67], and Brown et al. [68] reported the general reviews of cavity-enhanced techniques and their relative performance. Cavity ring-down spectroscopy has become a widely used technique in the optical absorption analysis of atoms, molecules, and optical components.

**Figure 10:**
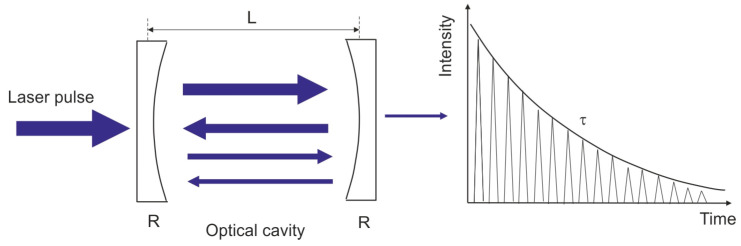
Idea of CRDS operation.

The principle of the CRDS method entails measuring the decay time of radiation trapped in an optical resonator. A pulsed laser beam with a duration of about 50 ns is introduced into a cavity consisting of two high-reflectance mirrors (R > 99.995%). After each reflection from the mirrors, a small portion (1 − R) of the circulating radiation is output while the remainder is reflected back into the cavity (Figure 10).

The radiation detection behind the output mirror is on a photomultiplier tube and monitored on the output mirror on a digital oscilloscope. If an absorbing element is placed in the cavity, the decay time of the laser pulse will be shortened at the wavelength of absorption of the absorbing gas. The radiation decay time is measured first with an empty cavity and then with the cavity filled with an absorber. The time of disappearance of radiation t in the cavity is expressed by the Formula (5):

(5) $$\tau =\frac{L}{c\left[1-R\right)+\alpha L]}$$

where: *L*—the length of the cavity, *R*—the reflectance of the mirrors, and *α*—absorption coefficient. 

Today, there are many techniques in which such a solution is used. The most commonly used are:

- P-CRDS (pulsed) method, in which pulsed lasers are used [69],
- CW-CRDS (Continuous Wave) method with continuous action lasers [70],
- CEAS method (Cavity Enhanced Absorption Spectroscopy) consisting of off-axis introduction of a radiation beam into the optical cavity [71],
- EW-CRDS method (Evanescent Cavity Ring-Down Spectroscopy), which uses the vanishing wave mechanism [72],
- F-CRDS (Fiber Cavity Ring-Down Spectroscopy) method [73],
- RSP method (Ring-Down Spectral Photography), i.e., a method with spectral decay photography [74].

Different variations of the CRDS method have been used to detect methane with ppb sensitivity [75,76,77,78]. 

Lang et al. [78] presented the operation of a single-mode distributed feedback ICL coupled to a V-shaped optical cavity (OF-CEAS). This arrangement reduces feedback to the laser from non-resonant reflections and ensures that the minimum detectable absorption coefficient is (7.1 ± 0.2) × 10^−8^ cm^−1^ for the CH_4_ spectrum at 3.24 mm. This corresponds to a detection limit of 3 ppb of CH_4_ at atmospheric pressure.

Kassi et al. [79] described the sensitive detection of atmospheric methane using a cascade quantum laser with feedback cavity-enhanced absorption spectroscopy (OF-CEAS). A noise-equivalent absorption coefficient of 3.6 × 10^−9^ cm^−1^ Hz^−1/2^ was achieved for the CH_4_ spectral scan at 7.39 μm, and the detection limit of 39 ppt CH_4_ at atmospheric pressure within a 50 s acquisition time was found. The OF-CEAS method was used to measure the atmospheric methane content in the air and to measure volcanic emissions in situ [79].

A technique known as cavity-enhanced frequency modulation spectroscopy or optical noise-resistant heterodyne spectroscopy (NICE-OHMS) was developed [80]. It was showed that methane was detected in a cell with an absorption coefficient α_min_ of 4 × 10^−11^ cm ^−1^ Hz^−1/2^ [81].

## 7. Remote Methane Detection Systems

An overview of spectroscopic methods for remote gas detection is presented in the article of Bogue [82], and remote methane detection is described in the work [83,84,85,86,87,88,89,90,91,92,93,94,95,96].

**Figure 11:**
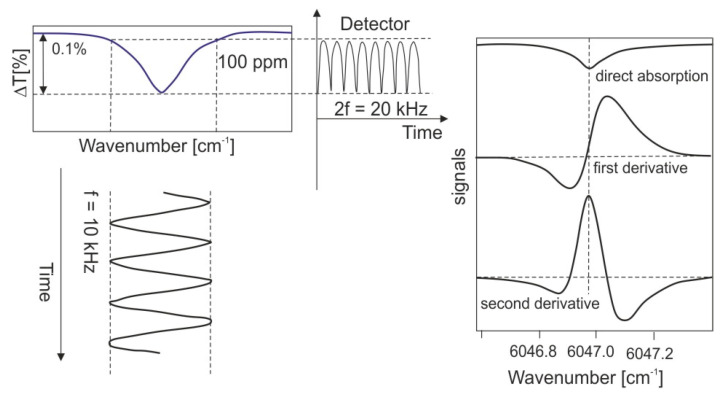
The phase-sensitive detection method.

The first portable TDLS remote detection system using a semiconductor diode with a wavelength of 1.65 μm was made by a Japanese group [49,50]. The product was implemented by Tokyo Gas Co. and is still in use. The device was made based on the frequency modulation (FM) of InGaAsP DFP diode radiation using the InGaAs-PIN photodetector. The sensor was designed as a handheld, portable absorption lidar with a range of up to 10 m. It has the capability to detect leakage at a speed of 10 mL/min or concentration of 100 ppm × m with a time constant of 0.1 s and at a distance of 1–10 m in real field conditions. The diode radiation is modulated by a sinusoidal change of current with a frequency of f = 10 kHz. The object receives the dispersed signal through the phase-sensitive detection method at the basic frequency as well as its second harmonic (2 f = 20 kHz) (Figure 11).

Ikuta et al. [52] developed a laboratory DIAL system for the remote detection of CH_4_ using a tunable parametric generator OPO (Optical Parametric Oscillator) based on a pulsed titanium–sapphire laser pumped with an Nd: YAG laser and a Raman shift. The energy at the output of the laser with a wavelength of 1.6659 (6002.56 cm^−1^) was 7.5 mJ. It enabled the detection at distances up to 500 m with a spatial resolution of about 15 m and detectability of 6000 ppm × m at a distance of 130 m.

**Figure 12:**
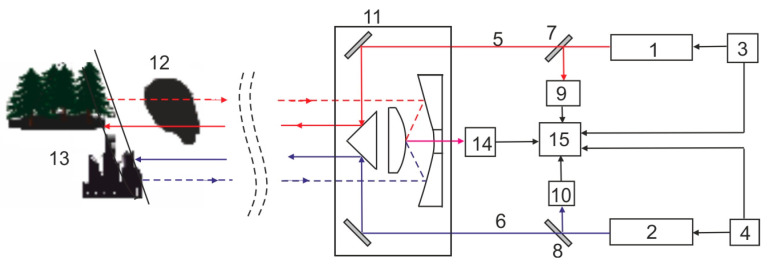
The diagram of the DIAL measuring system and its view.

At the Institute of Optoelectronics MUT, new solutions for measuring methane concentration remotely in the atmosphere [97] were developed and tested; methane absorption lines were adopted from the ν_3_ bands for monitoring. The probing beam (λ_on_) tuned to the P(7) line of methane was given by the He-Ne laser (3.39 μm) and the reference beam (λ_off_) was given by the He-Xe laser (3.51 mm). The use of lasers with a power of 30 mW, a cooled HgCdTe detector optimized for the received band, and the use of under-noise signal processing allow for estimation in the range of 100–200 m and the measurement of the concentration in the range of 50–2⋅10^5^ ppm. Transmission-type receiving optics with an input diameter of 8 cm and HgCdTe detector with a two-stage refrigerator were used. A measuring range of up to 50 m was achieved. The diagram of the DIAL measuring system, its method of operation, and its view are shown in Figure 12.

The He-Ne laser (1) generates a beam (5) (red arrows) with a wavelength of 3.39 μm, and the He-Xe laser (2) generates a beam (blue arrows) (6) with a wavelength of 3.51 μm. Both lasers are stimulated by a high-frequency current using switched-mode power supplies ((3) and (4)). Such a power supply allows for the pulsed amplitude modulation of laser beams. The beam (5) generated by the He-Ne laser is directed to the light-sharing plate (7), and the beam (6) generated by the He-Xe laser is directed to the light-sharing plate (8). Both light plates divide a small part of the radiation of both beams (5) and (6) direct it to auxiliary detectors (9) and (10). Then, they transmit the remaining part of the radiation with much higher energy towards the optical mirrors of the transceiver system (11). With the optical elements of the transceiver system, the radiation is directed to the studied region of the atmosphere (12), which may contain methane. After passing through this area, the radiation is reflected and scattered by objects (buildings, trees) (13) in its path. The reflected radiation returns to the transceiver (11) and is directed through its optical elements to the main receiving detector (14). From the detector, the signal is transmitted to the electronic signal analysis system (15). Signals from the output of auxiliary detectors (9) and (10) are also directed to this system. These signals act as reference signals. In the electronic signal analysis system, the value of the difference in the radiation intensity of the signals received by the receiver is calculated. The active detection system is designed to remotely detect the presence of methane in the atmosphere and the location of its leakage site.

An interesting solution is up-conversion range-resolved DIAL (Differential Absorption LIDAR) for measuring atmospheric methane [97,98]. The compact and powerful up-conversion detector (UCD) is built on an in-cavity pump system. The 1064 nm laser radiation is mixed with the lidar backscatter signal (1646 nm) in a periodically polarized lithium niobate crystal, and the up-conversion signal at 646 nm is detected by PMT. The UCD noise equivalent power is about 127 fW/Hz^1/2^ and surpasses the conventional InGaAs avalanche photodetector.

Global spatial and temporal measurements of atmospheric methane concentrations are carried out as part of Methane Remote Sensing Lidar, The Mission MERLIN [98]. The project is a collaboration between France and Germany and is aimed to launch and operate the CH_4_ monitoring satellite.

For the first time, the atmospheric composition was measured from space using an IPDA (Integrated Path Differential Absorption) LIDAR. The IPDA technique relies on DIAL (Differential Absorption LIDAR) measurements using a pulsed laser emitting at two wavelengths. This instrument measures the light that is scattered and reflected from the Earth’s surface and cloud tops which are illuminated by laser pulses with slightly different wavelengths: λ_on_ (1.16455 mm) and λ_off_ (1.16458). The online wavelength λ_on_ is positioned on one of the CH_4_ absorption lines at 1.64 µm. The measurement at λ_off_ serves as the reference measurement with negligible absorption by the CH_4_ molecules in the path.

One of the main anthropogenic sources of methane are landfills. Biogas, which consists mainly of methane, is produced by anaerobic degradation of waste. Methane emitted into the atmosphere also comes from leaking wells and leaks in gas collection systems. DIAL is an important remote sensing technique that can be used to track methane clouds emitted from petrochemical plants, wells from landfills, waste treatment plants, and wastewater. 

For more than 25 years, more than 70 industrial sites in eleven different countries have used the National Physical Laboratory (NPL) system [99]. The system has been used to test CH_4_ emissions from landfills at more than 30 sites in the UK, France, and the USA. Measurements were made of methane emissions from active, closed areas and gas engine piles. A comparative study of five methods for measuring methane emissions in the field was carried out at French, England, and Wales landfills [100]. 

The combination of TDLS and imaging for the sensitive and quantitative visualization of gas leaks opens up new possibilities. For example, in the article [101], the detection of methane leakage in the use of an interband cascade laser (3.27 μm) and an infrared camera is presented. Methane leakage was visualized at a rate of up to 2 mL/min in successive image sequences at frame rates up to 125 Hz. The gas cloud and leakage were localized and quantified in a single image. 

Instruments manufactured by Aerodyne Res. were used to study CH_4_ emissions from offshore oil and gas platforms in the Gulf of Mexico [102]. The TILDAS measuring system was equipped with a three-tunable infrared laser and a custom 400-m multipass absorption cell. It was possible to detect methane in the ambient air at the ppb level.

## 8. Photoacoustic Detection 

PAS of gases has been covered in reviews [103,104]. The photoacoustic phenomenon which entails the formation of an acoustic signal in the medium under the influence of variable intensities of optical radiation was discovered as early as 1880. The phenomenon of selective absorption of electromagnetic radiation by the analyzed substances is used here. The absorption of radiation energy is accompanied by an increase in the temperature and, as a result, there is a change in the sound pressure inside the analyzed gas chamber. Heating the gas with the absorption of the modulated intensity of incident radiation is also a cyclic process, so we can talk about a periodic heat flux.

If the radiation beam is interrupted at a frequency below 10 kHz, periodic pressure changes occur in the absorption cell, which can be detected using a sensitive microphone placed on the cell wall. The output signal from the microphone is proportional to the energy absorbed by the radiation, so it can be used to determine the concentration of the determined gas. In the simplest flow-through optoacoustic detector, the determined gas flows through a measuring chamber where it is irradiated with modulated infrared radiation and a specific wavelength strictly for this gas lying in the area of the absorption band. The detector is calibrated with a sample of gas measured at a precisely known concentration; it is the same detector being used to detect different gases. Before the measurement, the interference filter is replaced with one characteristic of the currently marked gas (e.g., filter carousel). 

**Figure 13:**
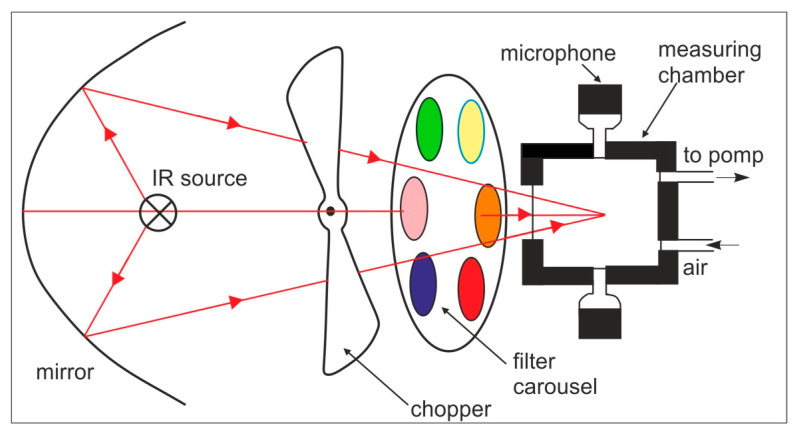
Diagram of the Innova 1312 photoacoustic analyzer.

The custom-built Inova 1312 photoacoustic analyzer for the determination of five selected gases and water vapors is shown in Figure 13.

It uses a light source operating in the mid-infrared range. The light beam passes through the modulator and the filter carousel. Each of the filters passes a narrow band of wavelengths, characteristic of a specific chemical compound. The specified gas in the measuring chamber causes the absorption of incident IR radiation to a degree directly proportional to its concentration. The analysis of individual gases is realized by selecting an appropriate selective gas absorption band in the mid-infrared range and matching a narrowband interference filter covering the given band.

The accurate analysis of methane is therefore possible in the absence of other hydrocarbons (e.g., gas pipelines, landfills). In other cases, only the so-called total hydrocarbon content (expressed per CH-group) can be determined. A reliable analyzer serves as a comparison system in field research. The measurements are characterized by high precision. Sensor systems based on PAS are very effective tools for detecting trace gases [105,106,107]. Their design is compact and economical, but they are sensitive to mechanical and acoustic vibrations. 

When replacing the microphone with a commercially available, inexpensive quartz tuning fork (QTF), a much better performance is achieved. This technique is called quartz-enhanced photoacoustic spectroscopy (QEPAS). A quartz tuning fork (QTF) provides resistance to external noise. The piezoelectric current generated by the tuning fork is detected by means of a transimpedance amplifier. The minimum normalized absorption coefficients can be achieved at the level of αmin in the range of 10^−8^–10^−9^ cm^−1^ W Hz^−1/2^ [108,109]. To improve the sensitivity, several research groups have developed microcantilevers as alternatives to microphones. They are characterized by very low mechanical susceptibility, show high deflections to pressure waves, and low mechanical susceptibility [110,111].

## 9. Summary

The issue of methane detection has a very long history, but it is still relevant. This article presents practically used instruments and optical methods for detecting this gas. Different techniques with different sensitivities are used to detect and determine methane concentrations. To determine methane in the upper atmosphere or in human breath, instruments are necessary to detect it based on their levels of sensitivity to ppb. In mines and residential buildings where natural gas is used, sensors with a much lower sensitivity of 0.1% are used. An important problem is the detection of methane leaks on transmission lines or the search for natural gas sources. Remote detection techniques are used here. Over the past 20 years, with the advent of mid-infrared cascade lasers, a revolution has been made in the construction of sensitive methane analyzers that are able to detect arbitrarily low concentrations. This article also presents examples of instruments developed at the Institute of Optoelectronics of the University of Technology, including the non-dispersion and non-dispersion analyzers, or optical correlators, TDLAS and DIAL systems.

The topic of methane detection in various applications is still valid. The TDLS technique has made it possible to detect this gas in the upper atmosphere and in the breath at the ppb level, and there is no need to look for more sensitive methods. There are currently commercially available QCL and ICL lasers, and highly sensitive infrared detectors and optics. In the range of higher methane concentrations on the order of 0.1%, techniques such as correlation spectroscopy have been developed, which allow for the quantitative analysis of methane in the presence of other hydrocarbons. 

This prevents, for example, false alarms caused by leaks of butane–propane mixtures. This is a very promising direction for the development of methane analyzers. The detection of methane leakages is still a current problem. One of the promising solutions has recently been the use of an unmanned aerial vehicle (UAV), equipped with a remote sensing methane detector, for natural gas leak detection from the pipeline network [112,113].

An example of how serious the problem of methane growth in the air can be is seen in the damage to the Nord Stream 2 pipeline and the leakage of a huge amount of gas into the atmosphere. Methane measurements usually cover a very small area. So far, there are no solutions for systems covering larger areas, e.g., coal mines or entire cities, in the form of connected separate multiple sensors. An important undertaking is the replacement of pellistors with optical sensors in coal mines. It is still a big challenge, because air pollution with coal dust complicates the introduction of optical methods.

## Figures and Tables

**Table 1:** Technical data of methane analyzer built from opto-pair (RMt Ltd.).

Parameters	Values
Concentration range	0–2%
Resolution	10 ppm
Absolute error [ppm]	± (50 + 0.05 C)
Scale drift	0.2%
Analysis time	<40 s
Dimensions [mm]	150 × 80 × 60
Weight	300 g

**Table 2:** The parameters of CIPS analyzer.

Parameters	Values
Concentration range [ppm]	1–10,000
Sensitivity [ppm]	0.1
Relative error [%]	± 5
Selectivity	methane-butane 4000:1
Measuring time [s]	3
Dimensions [mm]	400 × 240 × 200
Weight	4 kg

## Data Availability

Not applicable.
